# Different effects of age, adiposity and physical activity on the risk of ankle, wrist and hip fractures in postmenopausal women

**DOI:** 10.1016/j.bone.2012.03.014

**Published:** 2012-06

**Authors:** Miranda E.G. Armstrong, Benjamin J. Cairns, Emily Banks, Jane Green, Gillian K. Reeves, Valerie Beral, Million Women Study Collaborators

**Affiliations:** a Cancer Epidemiology Unit, University of Oxford, Oxford, UK; b National Centre for Epidemiology and Population Health, The Australian National University, Canberra, Australia

**Keywords:** Hip fracture, Wrist fracture, Ankle fracture, Physical activity, BMI, Postmenopausal women

## Abstract

While increasing age, decreasing body mass index (BMI), and physical inactivity are known to increase hip fracture risk, whether these factors have similar effects on other common fractures is not well established. We used prospectively-collected data from a large cohort to examine the role of these factors on the risk of incident ankle, wrist and hip fractures in postmenopausal women. 1,155,304 postmenopausal participants in the Million Women Study with a mean age of 56.0 (SD 4.8) years, provided information about lifestyle, anthropometric, and reproductive factors at recruitment in 1996–2001. All participants were linked to National Health Service cause-specific hospital records for day-case or overnight admissions. During follow-up for an average of 8.3 years per woman, 6807 women had an incident ankle fracture, 9733 an incident wrist fracture, and 5267 an incident hip fracture. Adjusted absolute and relative risks (RRs) for incident ankle, wrist, and hip fractures were calculated using Cox regression models. Age-specific rates for wrist and hip fractures increased sharply with age, whereas rates for ankle fracture did not. Cumulative absolute risks from ages 50 to 84 years per 100 women were 2.5 (95%CI 2.2–2.8) for ankle fracture, 5.0 (95%CI 4.4–5.5) for wrist fracture, and 6.2 (95%CI 5.5–7.0) for hip fracture. Compared with lean women (BMI < 20 kg/m^2^), obese women (BMI ≥ 30 kg/m^2^) had a three-fold increased risk of ankle fracture (RR = 3.07; 95%CI 2.53–3.74), but a substantially reduced risk of wrist fracture and especially of hip fracture (RR = 0.57; 0.51–0.64 and 0.23; 0.21–0.27, respectively). Physical activity was associated with a reduced risk of hip fracture but was not associated with ankle or wrist fracture risk. Ankle, wrist and hip fractures are extremely common in postmenopausal women, but the associations with age, adiposity, and physical activity differ substantially between the three fracture sites.

## Highlights

► Risk factors for ankle, wrist, and hip fractures differ by age, adiposity, and physical activity in postmenopausal women. ► Age-specific rates for wrist and hip fractures increased sharply with age, whereas rates for ankle fracture did not. ► Obese women had a three-fold increased risk of ankle fracture, when compared with lean women. ► Compared with lean women, obese women had a 43% and 77% reduced risk of wrist and hip fractures, respectively. ► Physical inactivity was associated with increased risk of hip fracture, but had little association with ankle or wrist fracture.

## Introduction

The incidence of hip fracture rises steeply with age. We and others have reported previously that hip fracture risk is decreased with increasing body mass index (BMI) and with physical activity [1–4]. Increasing BMI is associated with a reduced risk of hip fracture through three main mechanisms: an increased strain on the bones [5], greater adipose tissue leading to an enhanced ability to produce endogenous estrogens [6], and cushioning of bone by adipose tissue during a fall [7]. Physical activity may reduce fracture risk through improved muscle strength and balance, and by preservation of bone mass [8,9] but conversely the risk of injury may be increased while participating in physical activities [10]. There is limited evidence on the relation of BMI and physical activity to fracture risk at sites other than the hip. We describe here the relationships of age, BMI, and physical activity with the risk of ankle, wrist, and hip fractures in a large cohort of postmenopausal women in the UK (with extended follow-up since our previous report on hip fracture [1]). We also calculate age-specific and cumulative absolute risks for fracture of the ankle, wrist, and hip for postmenopausal women from ages 50 to 85 years.

## Methods

### Participants and data

The Million Women Study is a large prospective cohort study of women in the UK. Details of the design and methods of the study have been described elsewhere [11]. In short, 1.3 million women invited for breast cancer screening at National Health Service (NHS) clinics in England and Scotland were recruited into the study in 1996–2001 by completing a questionnaire, which included questions on anthropometry, physical activity, and other factors, and giving written consent to participate (see http://www.millionwomenstudy.org). Ethics approval was provided by the Oxford and Anglia Multi-Centre Research Ethics Committee.

Each woman's unique NHS identification number, together with other personal information, was used to link to cause-specific information on NHS hospital admission databases: Hospital Episodes Statistics for England, [12] and Scottish Morbidity Records in Scotland [13]. The databases include information both on inpatient (i.e. overnight) stays and day-case admissions (where women were admitted and discharged on the same day, e.g. for procedures such as the reduction of a fracture), but not on outpatient visits.

Information on the date of diagnoses and procedures associated with each hospital admission were provided, coded to the World Health Organisation's International Classification of Diseases, 9th and 10th revisions (ICD-9 and ICD-10) [14] for diagnoses and the Office of Population Censuses and Surveys' classification of surgical operations and procedures, fourth revision (OPCS-4) [15] for procedures. Incident cases were defined as the first hospital record (day-case or overnight admission) of ankle fracture (824.0–824.9, ICD-9; S82.3, S82.5–S82.6, S82.8, ICD-10), of wrist fracture (813.4, 813.5, 814.0–814.1 ICD-9; S52.5–S52.6, S62.0–S62.1, S62.8, ICD-10), or of hip fracture (820, ICD-9; S72.0–S72.2, ICD-10) occurring after recruitment into the study. For the purposes of censoring at the first occurrence of any fracture (see below), all other fractures were defined as codes: 800.0, 800.5, 801.0, 801.5, 802, 803.0, 803.5, 804.0 804.5, 805, 807–829 (ICD-9) and M48.4, M80, M84.3, S02, S12, S22, S32, S42, S52, S62, S72, S82, S92, T02, T08, T10, T12, T14.2, X59.0 (ICD-10).

Analyses were restricted to postmenopausal women: those who reported at baseline that they had experienced natural menopause (49%), or had undergone a bilateral oophorectomy (6%) were defined as postmenopausal. Women who were premenopausal, perimenopausal, or of unknown menopausal status at recruitment, were assumed to be postmenopausal after they reached the age of 55 years, as 96% of women in this cohort with a known age at natural menopause were postmenopausal by that age. Women who had a hospital record with a fracture prior to recruitment, had a diagnosis of cancer prior to recruitment; and those who reported at recruitment having been treated for osteoporosis or having had a prior stroke were excluded from analyses. These exclusions were applied as these conditions might affect subsequent weight and physical activity, bone mineral density and the propensity to fall [16,17].

### Measures of body size and physical activity, and other factors

At recruitment women were asked to report their height measured in feet and inches and their weight measured in stones and pounds. Heights were converted to the nearest 1 cm and weights to the nearest 0.1 kg, and this information was used to calculate BMI as weight (kg)/height (m)^2^. To assess the combined effects of measurement error and changes in women's BMI over the follow-up period, a sample of women was asked to have their weight and height measured by their general practitioners 9 years after their reporting of height and weight. We used this information from 2772 women eligible for the present study to compare BMIs calculated from self-reported data at baseline to BMIs calculated from measured data 9 years later and found excellent agreement (correlation coefficient = 0.85) [1].

Frequency of strenuous activity was assessed by asking, “How often do you do any strenuous exercise? (that is, enough to cause sweating or a fast heart beat)” and frequency of any physical activity by the question, “How often do you do any exercise?”, each with the options: Rarely/never, less than once a week, once a week, 2–3 times a week, 4–6 times a week, every day. The first 9% of the questionnaires did not ask the question on frequency of “any” physical activity. Ability of these questions to discriminate between different activity levels in this population was assessed by comparing responses to these questions with excess metabolic-equivalent hours (MET-hours). MET-hours were estimated from reported time spent walking, gardening, cycling, and doing strenuous activity about 3 years later (first resurvey), according to Ainsworth's compendium of physical activities [18,19]. Wareham et al. [20] has shown that the self-reported number of hours spent cycling, doing strenuous activity, and occupational activity is positively associated with objective physical activity measures. We did not include occupational activity in our analyses as only 20% of women reported being in full-time work at first resurvey [19].

Approximately 3 and 7 years after recruitment women were resurveyed. On these questionnaires they were asked: “In the last 5 years have you had any broken/fractured bones?” and if they answered “yes”, they were then asked to report whether their most recent fracture had resulted from a fall.

### Analysis

The statistical package Stata, version 10.1 [21] was used for all analyses. Person-years were calculated from the date of recruitment. For women in Scotland, hospital data was available from January 1, 1981. In England the first date for reliable hospital data was April 1, 1997 and follow-up was calculated from that date for the 5% of women recruited prior to then. Follow-up was censored at whichever came first of: the date of any fracture; date of death; date of emigration; or the end of follow-up. For participants in England, the last date of follow-up was March 31, 2008; and for participants in Scotland the last date of follow-up was December 31, 2008.

Cox regression models with attained age as the underlying time variable were used to estimate relative risks (RR) and 95% confidence intervals for incident ankle, wrist, and hip fractures by BMI and physical activity. Analyses were stratified by recruitment region (ten regions) and adjusted for: socio-economic status (quintiles using the Townsend index [22]), smoking status (current, past, never), alcohol consumption (0, 1–2, 3–6, 7–14, ≥ 15 drinks per week), menopausal hormone therapy use (never, past, current), diabetes (yes, no), history of heart disease/thrombosis (yes, no), history of osteo/rheumatoid arthritis (yes, no), thyroid disease (yes, no), and height (< 155, 155.0 to 159.9, 160 to 164.9, 165.0 to 169.9, or ≥ 170 cm). Depending on the model, additional adjustments included: BMI (< 20, 20.0–22.4, 22.5–24.9, 25.0–27.4, 27.5–29.9, ≥ 30.0 kg/m^2^), and strenuous physical activity (rarely/never (inactive), at most once per week, or more than once per week). Missing data for the adjustment variables (generally < 2% for each variable) were assigned to an additional category. The RRs were treated as floated absolute risks [23] when more than two categories were used for risk comparisons, and given with corresponding floated confidence intervals (FCIs), so that valid comparisons can be made between any two groups. When only two categories are compared or when log-linear trends in risk are quoted, conventional confidence intervals are used. To ensure that the impact of measurement error was minimised, category specific relative risks based on self-reported data were plotted against mean measured BMI values within each category.

Age-specific incidence rates per 100 women over 5 years were calculated for each fracture site for 5-year age groups from 50–54, to 80–84 years. Cumulative risks from ages 50 to 85 were calculated for each fracture site, taking the average hazard rate over this time period to be the uniformly age-standardised incidence rate per person-year. Cumulative absolute incidence rates for women aged from 50 to 79 were also calculated for each fracture site according to BMI and strenuous physical activity categories. To allow for potential non-proportional hazards, such as might be associated with the dramatic increase in incidence of hip fractures with age, we analysed the data in 10 year age bands. For each fracture type and exposure, category-specific relative risks were converted to incidence rates by multiplying them by the appropriate age-specific incidence rate, divided by a weighted average of all relative risks [24]. These incidence rates were age-standardised across the full age range from 50 to 79 and used to compute cumulative risks as above. Possible interactions between the associations of BMI and physical activity, and use of menopausal hormone therapy were assessed using likelihood ratio tests.

## Results

**Fig. 1:**
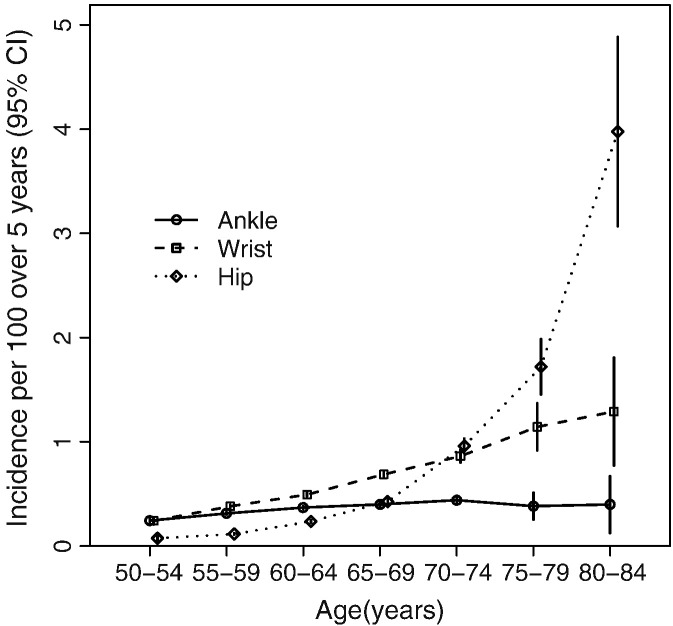
Age-specific incidence per 100 over 5 years (95%CI) of ankle, wrist, and hip fractures among post-menopausal women.

The baseline characteristics of the 1,155,304 postmenopausal women included in these analyses are shown in Table 1. Women were on average 56.0 (SD 4.8) years of age at recruitment, with a mean BMI of 26.2 (SD 4.7) kg/m^2^ at recruitment. Mean BMI decreased and mean alcohol consumption increased with increasing frequency of physical activity. During a mean follow-up of 8.3 years per woman (almost 10 million person-years), 6807 women had an incident ankle fracture, 9733 had an incident wrist fracture, and 5267 had an incident hip fracture. Our previous report, with shorter follow-up, included only 2582 women with an incident hip fracture [1]. Age-specific incidence rates did not vary much for ankle fracture, but rates increased gradually with age for wrist fracture and very steeply with age for hip fracture (Fig. 1 and eTable 1). The estimated cumulative absolute risks per 100 women from ages 50 to 84 years were 2.5 (95%CI 2.2–2.8) for ankle fracture, 5.0 (95%CI 4.4–5.5) for wrist fracture, and 6.2 (95%CI 5.5–7.0) for hip fracture.

**Fig. 2:**
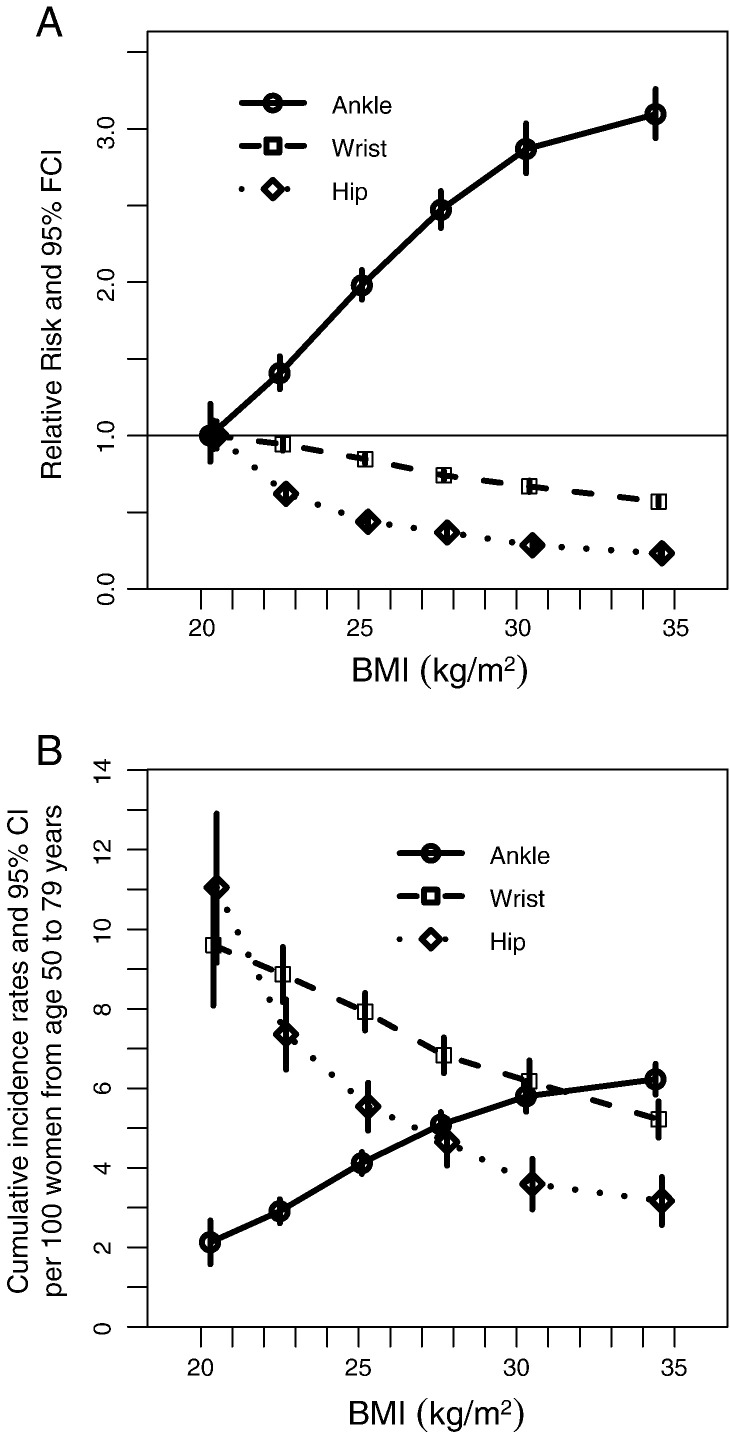
Adjusted relative risks for all women (A) and adjusted cumulative incidence rates from ages 50 to 79 years (B) of ankle, wrist and hip fractures in post-menopausal women, by BMI. *Risk estimates are plotted against the mean measured BMI value within each category*.

Having a higher BMI was associated with an increased risk of ankle fracture, and a reduced risk of wrist and hip fractures, over the full study age range (Fig. 2 and Table 2). Compared with lean women (BMI of < 20.0 kg/m^2^), for women of normal weight (BMI 20.0–24.9 kg/m^2^) the RR for ankle fracture was 1.77 (95%CI 1.46–2.14), for overweight women (BMI 25.0–29.9 kg/m^2^) the RR was 2.62 (95%CI 2.16–3.17), and for obese women (BMI of ≥ 30.0 kg/m^2^) the RR was 3.07 (95%CI 2.53–3.74). Compared with lean women the RR for wrist fracture was 0.88 (95%CI 0.80–0.97) in normal weight women, 0.71 (95%CI 0.65–0.79) in overweight women, and 0.57 (95%CI 0.51–0.64) in obese women. For hip fracture, the corresponding RRs were 0.51 (95%CI 0.46–0.56), 0.34 (95%CI 0.30–0.37) and 0.23 (95%CI 0.21–0.27). As there was a large increase in the incidence of hip fractures with age we also analysed the data in 10 year age bands. The relationship of BMI to hip and ankle fracture was weaker in women aged ≥ 70 than in younger women. In contrast, the BMI–wrist fracture relationship was stronger in older than in younger women (eTable 2).

The increase in risk of ankle fracture per five-unit increase in BMI among women with a BMI of < 25 kg/m^2^ was significantly greater than the increase per five-unit increase in BMI in overweight and obese women (RRs per 5 kg/m^2^ 1.96, 95%CI 1.71–2.24 versus 1.18, 1.12–1.24; p_heterogeneity_ < .001). The reduction in the risk of hip fracture per five-unit increase in BMI was also greater among normal and underweight women, than among overweight and obese women (RRs per 5 kg/m^2^ 0.46, 0.42–0.51 versus 0.71, 0.65–0.77; p_heterogeneity_ < .001). However there was no similar heterogeneity in the risks for wrist fracture (RRs per 5 kg/m^2^ = 0.84, 0.77–0.91 versus 0.83, 0.79–0.87; p_heterogeneity_ = .87).

**Fig. 3:**
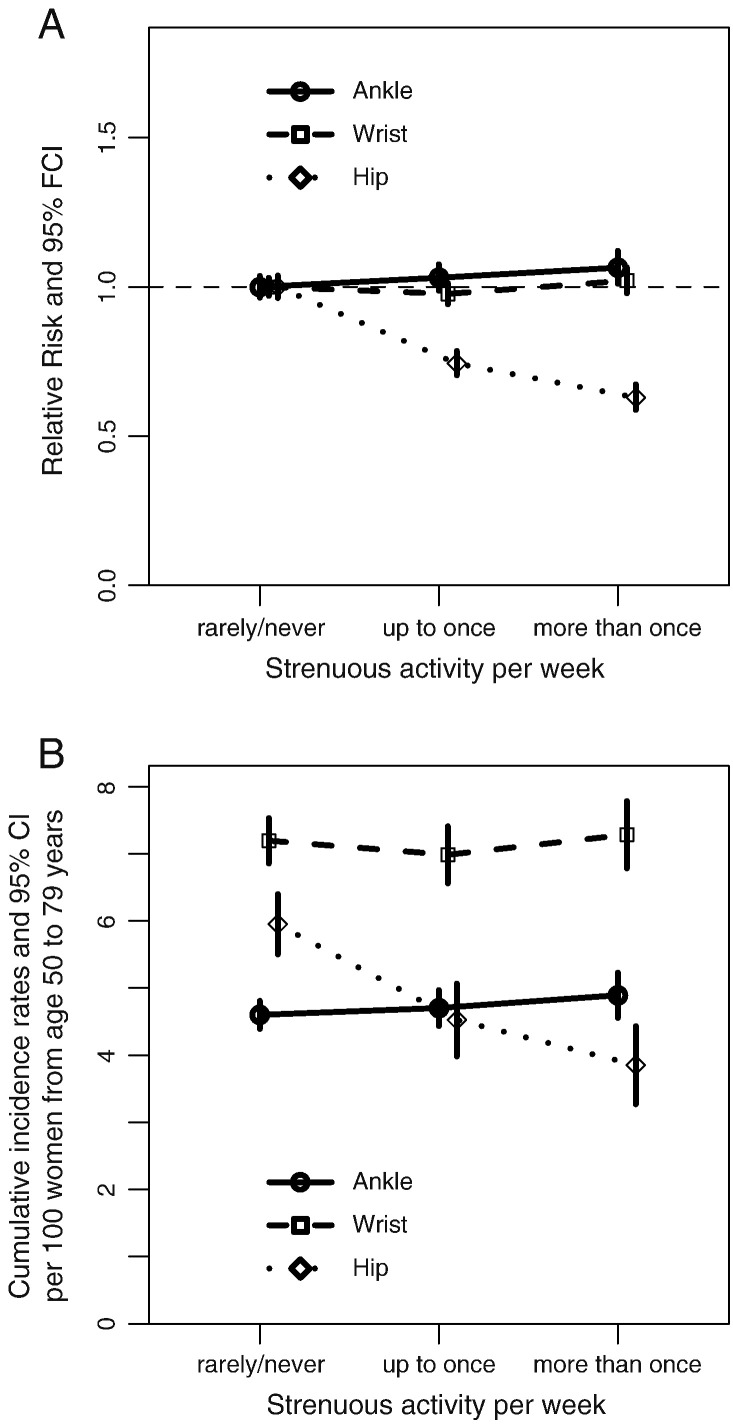
Adjusted relative risks for all women (A) and adjusted cumulative incidence rates from ages 50 to 79 years (B) of ankle, wrist and hip fractures in post-menopausal women, by strenuous physical activity.

No significant trends in the risk of ankle or of wrist fracture were observed by women's frequency of physical activity. By contrast, the risk of hip fracture was reduced substantially with increasing levels of both strenuous and any physical activity (Table 2 and Fig. 3).

Likelihood ratio tests suggested that there was no significant interaction between BMI and strenuous physical activity in association with ankle, wrist or hip fracture (p_interaction_ = .21, .42 and .77, respectively). There was also no significant interaction between BMI and any physical activity for ankle, wrist or hip fracture (p_interaction_ = .82, .83 and .18, respectively) (eTable 3).

A sensitivity analysis restricted to women without missing data for any of the adjustment variables showed similar risk relationships, as did a sensitivity analysis which excluded the first 3 years of follow-up (eTable 4).

The relationships between BMI and wrist and hip fractures did not vary significantly according to a woman's use of menopausal hormone therapy (p_interaction_ = .19 and .06, respectively; see eTable 5). The relation of BMI to ankle fracture was slightly, but significantly, stronger in current users of menopausal hormone therapy than in never users (p_interaction_ = .003; see eTable 5). The relation of strenuous activity to ankle, wrist, and hip fractures did not vary significantly by use of menopausal hormone therapy (p_interaction_ = .45, .93, and .34, respectively; see eTable 5). Nor was there any significant variation by menopausal hormone therapy use for any physical activity in relation to ankle or wrist fracture (p_interaction_ = .64 and .54). However, there was a smaller reduction in hip fracture risk associated with any physical activity in current users than in never users of menopausal hormone therapy (p_interaction_ = .007).

## Discussion

In this prospective study of 1.2 million postmenopausal women, 6807 had a record of one or more ankle fractures, 9733 had a record of one or more wrist fractures, and 5267 had a record of one or more hip fractures during a follow-up of about 8 years per woman. The cumulative absolute risk for ages 50 to 84 was 2.5% for ankle fracture, 5.0% for wrist fracture, and 6.2% for hip fracture. Age-specific rates for ankle fracture did not vary much, but rates for wrist fracture increased slightly, and rates for hip fracture increased sharply with age. We also found that the association with adiposity varied by fracture site. Increasing adiposity was associated with an increased risk of ankle fracture but a reduced risk of wrist and hip fractures. Trends in fracture risks per unit change in BMI tended to be greatest among lean women. Physical inactivity was associated with an increased risk of hip fracture, but had no material influence on risk of ankle and wrist fractures. The relationships of BMI and physical activity to fracture risk were independent of one another for ankles, wrists, and hips.

Prospective studies (including ours) have clearly established that there is a decreased risk of hip fracture among overweight and obese women [1,25,26]. However, the relationship between BMI and wrist and ankle fracture risk has been less clear, and this is the largest prospective study to examine these relationships in postmenopausal women. For ankle fractures, our findings of an increased risk with increasing adiposity are consistent with results from two retrospective case–control studies, [27,28] a retrospective cross-sectional study, [29] and two prospective studies;[30,31] however results from another prospective study were null [32]. For wrist fracture mixed findings have been reported, with the findings from two case–control studies consistent with a reduction in risk with increasing adiposity, [27,33] but no significant association was reported in two other case–control studies and in two prospective studies [32,34–36].

Physical activity has previously been associated with a reduced risk of hip fracture [1,25,37,38]. Published findings are mixed for fractures at other sites, and comparisons across studies are limited by the variation in the methods used to describe physical activity. For wrist fracture risk, some have reported that higher levels of physical activity were associated with an increased risk [32,39]; findings from another study showed no association with leisure-time physical activity [34]. In the Study of Osteoporotic Fractures cohort, wrist fracture risk varied by the type of physical activity [38,40]. For ankle fracture risk, in two prospective studies, higher levels of vigorous physical activity were associated with an increased risk in one study [41] but not in another [32].

The strength of this study lies in the large study population, its prospective nature, and the virtually complete follow-up for hospital records in the entire cohort. A limitation is the lack of a measure of bone mineral density [26]. Both peripheral and central bone mineral density have been shown to be associated with wrist and hip fractures [37,40,42–49] but not so strongly with ankle fracture [31,41–43,46]. Also, fractures not leading to day-case or overnight admission were not included in this study. Almost all hip fractures result in an overnight hospital stay, and most reduction procedures and/or anaesthetics given in relation to a wrist and ankle fracture would result in a day-case or overnight stay. Nevertheless, some relatively minor fractures may not be included in hospital data [50]. Our results show slightly lower incidence rates for hip fracture, and moderately lower incidence rates for ankle and wrist fractures than those reported in other UK studies [51–53]. However, the absolute rates quoted here are not directly comparable with those published previously since our analyses have excluded women with self-reported osteoporosis and other prior morbidities to exclude the possibility that these conditions altered behaviour. We also censored women at the first occurrence of any fracture (to account for the increased risk of subsequent fracture reported among women with a prior fracture [17]).

Falls are the most common reason for a fracture in the age group examined [54]. On a follow-up questionnaire about 7 years after recruitment, women who reported having had a fracture were asked how it occurred; over 85% of ankle, wrist, and hip fractures were associated with a fall. The fracture site associated with a fall is strongly dependent on the site of impact and the orientation of the fall [55,56]. Increased adiposity cushions the impact force for some bones, and this may be particularly relevant for hip fracture [7]. However, ankle fractures usually occur following rotation of the talus within the mortise, and higher torques are likely to result from twisting of the ankle in heavier than in lighter women [31]. Peripheral fat is the most important source of endogenous estrogen in postmenopausal women [57,58] and this increases bone mineral density [6]. In this cohort, the more obese women were, the more often they fell, [1] hence our results suggest that for ankle fracture, the effects of falls associated with obesity outweigh any beneficial effects of obesity on bone mineral density.

Physical activity has been hypothesised to have multiple opposing effects on fracture risk. It may decrease fracture risk, by maintaining bone mineral density and reducing bone loss, [8,9] and may protect against falls through improvement in balance, coordination and muscular strength [4]. However, during physical activity the individual may be at an increased risk of falls and injury, [10] and different types of activities may affect fracture risk in different ways. Physical activity had little influence on the risk of ankle and wrist fractures in our study, and it seems plausible that the competing factors associated with physical activity which act to increase and decrease the risk of fractures may balance each other out for these fracture types. Fracture risk is increased among frail individuals with multiple morbidities;[59] these individuals may also participate in less physical activity and may even have a low BMI as a result of their illness. Despite adjustment for a number of relevant illnesses and the consistency of findings following omission of the first 3 years of follow-up, we cannot exclude the possibility that part of the higher risk of hip fracture associated with physical inactivity and low BMI may be due to reverse causation.

In conclusion, risk factors for ankle, wrist, and hip fractures differ. Overweight and obese women were at a lower risk of wrist and particularly of hip fracture but a higher risk of ankle fracture when compared with lean and normal weight women. Physical inactivity was associated with an increased risk of hip fracture, but had little association with ankle or wrist fracture.

## Author contributions

ICMJE criteria for authorship read and met: MEGA BJC EB JG GKR VB. MEGA had full access to all data in the study and takes responsibility for the integrity of the data and the accuracy of the data analysis. Agree with the manuscript's results and conclusions: MEGA BJC EB JG GKR VB. Designed the experiments/the study: MEGA BJC EB JG GKR VB. Analysed the data: MEGA BJC. Collected data/did experiments for the study: VB EB. Enrolled patients: VB EB. Wrote the first draft of the paper: MEGA. Contributed to the writing of the paper: BJC EB JG GKR VB. Co-principal investigators of the Million Women Study: VB JG GKR.

## Competing interests

The authors have no competing interests to declare.

## Figures and Tables

**Table 1:** Characteristics of post-menopausal women in the Million Women Study at recruitment and follow-up according to BMI and strenuous physical activity a.

	BMI (kg/m^2^)						Strenuous exercise			All women
	< 20.0	20.0–22.4	22.5–24.9	25.0–27.4	27.5–29.9	30 +	Rarely/never (inactive)	At most once per week	More than once per week	
**Characteristics at baseline**	n = 41 818	n = 180 331	n = 313 534	n = 251 549	n = 162 477	n = 205 595	n = 553 040	n = 356 539	n = 245 725	n = 1 155 304
Mean age at recruitment, years (SD)	55.8 (4.9)	55.6 (4.8)	55.9 (4.8)	56.3 (4.8)	56.3 (4.8)	56.1 (4.7)	56.4 (4.9)	55.7 (4.7)	55.8 (4.7)	56.0 (4.8)
Mean height, cm (SD)	164.0 (7.1)	163.4 (6.4)	162.6 (6.4)	161.7 (6.5)	161.4 (6.6)	160.5 (7.0)	161.6 (6.7)	162.4 (6.5)	162.5 (6.6)	162.0 (6.7)
Mean weight, kg (SD)	50.9 (4.9)	57.2 (4.8)	62.9 (5.2)	68.5 (5.7)	74.6 (6.4)	87.6 (11.7)	70.1 (13.5)	68.3 (11.8)	66.8 (11.3)	68.8 (12.6)
Mean BMI, kg/m^2^ (SD)	18.9 (0.9)	21.4 (0.7)	23.7 (0.7)	26.1 (0.7)	28.6 (0.7)	34.0 (3.8)	26.9 (5.0)	25.9 (4.3)	25.3 (4.1)	26.2 (4.7)
Mean alcohol, grammes/day (SD)	6.7 (8.2)	7.3 (8.0)	7.0 (7.8)	6.4 (7.5)	5.8 (7.3)	4.7 (6.8)	5.6 (7.4)	6.7 (7.4)	7.4 (8.2)	6.3 (7.6)
Mean number of children (SD)	1.9 (1.4)	2.0 (1.2)	2.1 (1.2)	2.2 (1.3)	2.3 (1.3)	2.4 (1.4)	2.2 (1.3)	2.1 (1.2)	2.1 (1.3)	2.2 (1.3)
Current smoker (%)	31.8	22.9	20.0	19.0	18.2	16.0	24.4	15.4	15.6	19.7
Socioeconomic status: lowest fifth (%)	19.1	15.3	15.9	18.3	20.9	25.6	23.9	14.1	14.5	18.9
Never users of hormone therapy (%)	50.4	48.4	48.2	48.7	49.6	53.4	50.4	48.7	48.5	49.5
No physical activity (%)	20.1	15.6	16.7	20.0	24.2	31.9	42.7	1.2	1.2	21.1
No strenuous activity (%)	47.1	40.5	42.6	47.3	52.2	59.9	–	–	–	47.9

**Follow-up for fracture incidence**										
Woman-years of follow-up (in millions)	0.34	1.48	2.60	2.10	1.35	1,69	4.58	2.94	2.04	9.56
Incident ankle fractures (n)	109	660	1620	1614	1204	1600	3295	2068	1444	6807
Incident wrist fractures (n)	442	1800	2892	2079	1224	1296	4654	2910	2169	9733
Incident hip fractures (n)	497	1168	1451	1032	543	576	3149	1292	826	5267

a Women with missing values were excluded when calculating the means or percentages for that given variable.

**Table 2:** Effect of adjustment for various factors on the relative risks of ankle, wrist, and hip fractures in post-menopausal women by BMI and physical activity.

		Ankle fracture			Wrist fracture			Hip fracture		
	Population at risk n = 1,155,304	Incident cases n = 6807	Minimally adjusted b	Fully adjusted c RR (95% FCI a)	Incident cases n = 9733	Minimally adjusted b	Fully adjusted c RR (95% FCI a)	Incident cases n = 5267	Minimally adjusted b	Fully adjusted c RR (95% FCI a)
**BMI (kg/m^2^) (Mean measured)**										
< 20.0 (20.4)	41,818	109	1.00	1.00 (0.83–1.21)	442	1.00	1.00 (0.91–1.10)	497	1.00	1.00 (0.91–1.09)
20.0–22.4 (22.6)	180,331	660	1.39	1.41 (1.30–1.52)	1800	0.94	0.94 (0.90–0.99)	1168	0.56	0.62 (0.58–0.66)
22.5–24.9 (25.2)	313,534	1620	1.93	1.98 (1.89–2.08)	2892	0.84	0.85 (0.82–0.88)	1451	0.38	0.44 (0.42–0.46)
25.0–27.4 (27.7)	251,549	1614	2.37	2.47 (2.35–2.59)	2079	0.73	0.74 (0.71–0.77)	1032	0.32	0.37 (0.35–0.39)
27.5–29.9 (30.2)	162,477	1204	2.72	2.87 (1.71–3.04)	1224	0.66	0.67 (0.63–0.71)	543	0.26	0.28 (0.26–0.31)
30 + (34.5)	205,595	1600	2.90	3.09 (2.94–3.26)	1296	0.56	0.57 (0.54–0.60)	576	0.22	0.23 (0.21–0.25)
P-value (trend)				<.001			<.001			<.001

**Strenuous exercise**										
Rarely/never (inactive)	553,040	3295	1.00	1.00 (0.96–1.04)	4654	1.00	1.00 (0.97–1.03)	3149	1.00	1.00 (0.96–1.04)
At most once per week	356,539	2068	1.00	1.03 (0.99–1.08)	2910	1.03	0.98 (0.94–1.01)	1292	0.72	0.74 (0.70–0.79)
More than once per week	245,725	1444	1.01	1.06 (1.01–1.12)	2169	1.10	1.02 (0.98–1.07)	826	0.65	0.63 (0.59–0.67)
P-value (trend)				.05			.61			<.001

**Any exercise**	n = 1,045,666	n = 6094			n = 8737			n = 4606		
Rarely/never (inactive)	221,077	1293	1.00	1.00 (0.95–1.06)	1823	1.00	1.00 (0.95–1.05)	1252	1.00	1.00 (0.94–1.06)
At most once per week	251,069	1425	1.00	1.01 (0.96–1.07)	1952	0.99	0.95 (0.91–0.99)	937	0.73	0.78 (0.74–0.84)
2–3 times per week	248,279	1395	0.96	1.00 (0.95–1.06)	2088	1.01	0.94 (0.90–0.98)	912	0.65	0.67 (0.63–0.72)
More than 3 times per week	325,241	1981	1.02	1.11 (1.06–1.16)	2874	1.05	0.94 (0.91–0.98)	1505	0.78	0.72 (0.68–0.76)
P-value (trend)				.004			.08			<.001

a FCI = floating confidence interval.

b Adjusted for study region, age, and socio-economic status.

c Adjusted for study region, age, socio-economic status, smoking, alcohol consumption, parity, use of hormone therapy, height, heart disease/thrombosis, diabetes mellitus, thyroid disease, rheumatoid arthritis/osteoarthritis, and strenuous activity (for adjustment of BMI) or BMI (for adjustment of physical activity).
