# Supplementary material for: Different effects of age, adiposity and physical activity on the risk of ankle, wrist and hip fractures in postmenopausal women
Source: Bone. 2012 Jun;50(6):1394–400. doi: 10.1016/j.bone.2012.03.014 (PMC3405513; doi:10.1016/j.bone.2012.03.014)
Supplement: Supplementary file 1 — Supplementary materials. [file mmc1.doc]

**eTable 1:** Age specific incidence rates per 100 over 5 years for ankle, wrist and hip fractures in post-menopausal women

**eTable 2:** Relative risk of ankle, wrist, and hip fractures by BMI in post-menopausal women in 10-year age bands and BMI

**eTable 3:** Adjusted relative risks of ankle, wrist, and hip fractures in women, cross-classified by both BMI and strenuous physical activity

**eTable 4:** Sensitivity analyses for relative risk of ankle, wrist, and hip fractures in post-menopausal women according to BMI and physical activity

**eTable 5:** Sensitivity analyses of hormone therapy for relative risk of ankle, wrist, and hip fractures in post-menopausal women according to BMI and physical activity

| **eTable 1:** Age specific incidence rates per 100 over 5 years for ankle, wrist and hip fractures in post-menopausal women | | | | | | | | | | | |
| --- | --- | --- | --- | --- | --- | --- | --- | --- | --- | --- | --- |
|  |  |  |  | **ANKLE FRACTURE** | |  | **WRIST FRACTURE** | |  | **HIP FRACTURE** | |
|  |  | **Person-years** |  | **Incident**  **cases** | **Incidence rate per 100 (95% CI)** |  | **Incident cases** | **Incidence rate per 100 (95% CI)** |  | **Incident cases** | **Incidence rate per 100 (95% CI)** |
| **Age (years)** |  |  |  |  |  |  |  |  |  |  |  |
| 50-54 years |  | 469 554 |  | 243 | 0.24 ( 0.21-0.28) |  | 261 | 0.24 (0.21-0.28) |  | 77 | 0.07 (0.05-0.10) |
| 55-59 years |  | 3 294 869 |  | 2089 | 0.31 ( 0.30-0.33) |  | 2552 | 0.38 (0.37-0.40) |  | 780 | 0.12 (0.11-0.12) |
| 60-64 years |  | 3 139 848 |  | 2311 | 0.37 (0.35-0.38) |  | 3090 | 0.49 (0.48-0.51) |  | 1465 | 0.24 (0.22-0.25) |
| 65-69 years |  | 1 965 931 |  | 1579 | 0.40 ( 0.38-0.42) |  | 2643 | 0.69 (0.66-0.71) |  | 1602 | 0.43 (0.41-0.45) |
| 70-74 years |  | 613 053 |  | 528 | 0.44 ( 0.40-0.49) |  | 1024 | 0.86 (0.80-0.93) |  | 1018 | 0.96 (0.89-1.03) |
| 75-79 years |  | 60 701 |  | 43 | 0.38 ( 0.25-0.51) |  | 126 | 1.14 (0.92-1.37) |  | 202 | 1.72 (1.45-1.99) |
| 80-84 years |  | 11 409 |  | 9 | 0.40 ( 0.12-0.67) |  | 30 | 1.29 (0.77-1.81) |  | 91 | 3.98 (3.07-4.88) |

| **eTable 2:** Relative risk of ankle, wrist, and hip fractures in post-menopausal women in 10-year age bands and BMI | | | | | | | | | | |
| --- | --- | --- | --- | --- | --- | --- | --- | --- | --- | --- |
|  |  | **ANKLE FRACTURE** | | | **WRIST FRACTURE** | | | **HIP FRACTURE** | | |
| **BMI (kg/m^2^)** | **Population at risk** | **Incident cases** | **Minimally adjusted ^a^** | **Fully**  **adjusted** **^b^**  **RR (95% CI)** | **Incident cases** | **Minimally adjusted ^a^** | **Fully**  **adjusted** **^b^**  **RR (95% CI)** | **Incident cases** | **Minimally adjusted ^a^** | **Fully**  **adjusted** **^b^**  **RR (95% CI)** |
| **50 – 59 years** | n=847 350 | n=2332 |  |  | n=2813 |  |  | n=857 |  |  |
| <20.0 | 31 095 | 41 | 1.00 | 1.00 | 129 | 1.00 | 1.00 | 95 | 1.00 | 1.00 |
| 20.0-22.4 | 137 867 | 235 | 1.30 | 1.33 (0.95 - 1.85) | 555 | 0.97 | 0.97 (0.80 - 1.18) | 217 | 0.52 | 0.59 (0.47 - 0.76) |
| 22.5-24.9 | 233 281 | 546 | 1.81 | 1.88 (1.37 - 2.59) | 867 | 0.90 | 0.91 (0.76 - 1.10) | 220 | 0.32 | 0.37 (0.29 - 0.47) |
| 25.0-27.4 | 180 825 | 507 | 2.19 | 2.34 (1.70 - 3.21) | 589 | 0.79 | 0.81 (0.67 - 0.98) | 168 | 0.31 | 0.36 (0.28 - 0.46) |
| *27.5-29.9* | 115 234 | 409 | 2.79 | 2.99 (2.17 - 4.13) | 331 | 0.70 | 0.72 (0.58 - 0.88) | 75 | 0.22 | 0.24 (0.18 - 0.32) |
| *30+* | 149 048 | 594 | 3.09 | 3.37 (2.45 - 4.63) | 342 | 0.55 | 0.57 (0.46 - 0.70) | 82 | 0.18 | 0.17 (0.13 - 0.24) |
| *P-value (trend)* |  |  |  | < .001 |  |  | < .001 |  |  | < .001 |
|  |  |  |  |  |  |  |  |  |  |  |
| **60 – 69 years** | n=1,024,159 | n=3890 |  |  | n=5733 |  |  | n=3067 |  |  |
| <20.0 | 36 178 | 57 | 1.00 | 1.00 | 243 | 1.00 | 1.00 | 307 | 1.00 | 1.00 |
| 20.0-22.4 | 157 725 | 369 | 1.49 | 1.49 (1.13 - 1.97) | 1020 | 0.97 | 0.97 (0.84 - 1.12) | 689 | 0.53 | 0.59 (0.51 - 0.67) |
| 22.5-24.9 | 278 022 | 918 | 2.03 | 2.06 (1.57 - 2.69) | 1665 | 0.86 | 0.86 (0.75 - 0.98) | 839 | 0.35 | 0.40 (0.35 - 0.45) |
| 25.0-27.4 | 224 641 | 952 | 2.52 | 2.60 (1.99 - 3.40) | 1241 | 0.76 | 0.76 (0.66 - 0.87) | 578 | 0.28 | 0.32 (0.28 - 0.37) |
| *27.5-29.9* | 145 410 | 701 | 2.83 | 2.95 (2.25 - 3.87) | 749 | 0.70 | 0.70 (0.60 - 0.81) | 323 | 0.24 | 0.26 (0.22 - 0.31) |
| *30+* | 182 183 | 893 | 2.93 | 3.08 (2.35 - 4.03) | 815 | 0.62 | 0.62 (0.53 - 0.71) | 331 | 0.19 | 0.20 (0.17 - 0.23) |
| *P-value (trend)* |  |  |  | < .001 |  |  | < .001 |  |  | < .001 |
|  |  |  |  |  |  |  |  |  |  |  |
| **70 – 79 years** | n=254 880 | n=571 |  |  | n=1150 |  |  | n=1220 |  |  |
| <20.0 | 8 634 | 11 | 1.00 | 1.00 | 67 | 1.00 | 1.00 | 87 | 1.00 | 1.00 |
| 20.0-22.4 | 35 350 | 52 | 1.17 | 1.19 (0.62 - 2.29) | 217 | 0.81 | 0.82 (0.62 - 1.07) | 234 | 0.68 | 0.74 (0.58 - 0.95) |
| 22.5-24.9 | 66 989 | 151 | 1.81 | 1.87 (1.01 - 3.45) | 351 | 0.69 | 0.70 (0.54 - 0.91) | 354 | 0.55 | 0.62 (0.49 - 0.79) |
| 25.0-27.4 | 58 934 | 154 | 2.10 | 2.21 (1.19 - 4.08) | 241 | 0.54 | 0.54 (0.41 - 0.71) | 258 | 0.45 | 0.51 (0.40 - 0.66) |
| *27.5-29.9* | 39 036 | 93 | 1.94 | 2.04 (1.09 - 3.82) | 142 | 0.48 | 0.48 (0.36 - 0.65) | 133 | 0.36 | 0.39 (0.30 - 0.51) |
| *30+* | 45 937 | 110 | 2.04 | 2.16 (1.15 - 4.03) | 132 | 0.39 | 0.39 (0.29 - 0.53) | 154 | 0.37 | 0.39 (0.30 - 0.51) |
| *P-value (trend)* |  |  |  | < .001 |  |  | < .001 |  |  | < .001 |
| *P-value* ^c^ *(heterogeneity)* |  |  |  | .002 |  |  | .004 |  |  | < .001 |

^a^ Adjusted for study region, age, and socio-economic status

^b^ Adjusted for study region, age, socio-economic status, smoking, alcohol consumption, parity, use of hormone therapy, height, heart disease/thrombosis, diabetes mellitus, thyroid disease, rheumatoid arthritis/osteoarthritis, and strenuous activity

^c^ P-value for heterogeneity of trends per unit BMI between 10-year age bands

| **eTable 3:** Adjusted ^e^ relative risks of ankle, wrist, and hip fractures in women, cross-classified by both BMI and strenuous physical activity | | | | |
| --- | --- | --- | --- | --- |
| **BMI (kg/m^2^)** | **Rarely/never active**  **RR (FCI**^a^**)**  **(mean measured bmi) (n**^b^**)** | **At most once per week**  **RR (FCI**^a^**)**  **(mean measured bmi) (n**^b^**)** | **More than once per week RR (FCI** ^a^**)**  **(mean measured bmi) (n**^b^**)** | **P for interaction** |
| **Ankle** |  |  |  |  |
| <20.0 | 1.00 (0.77 - 1.30) ^d^  (20.4) (56) | 0.83 (0.57 - 1.20)  (20.7) (27) | 0.84 (0.57 - 1.23)  (20.0) (26) |  |
| 20.0-24.9 | 1.60 (1.50 - 1.71)  (24.5) (947) | 1.53 (1.42 - 1.65)  (24.1) (705) | 1.73 (1.60 - 1.87)  (24.1) (628) | .21 ^c^ |
| 25.0-29.9 | 2.31 (2.19 - 2.44)  (28.7) (1,356) | 2.43 (2.28 - 2.60)  (28.6) (893) | 2.43 (2.24 - 2.64)  (28.6) (569) |  |
| *30+* | 2.70 (2.52 - 2.88)  (34.9) (936) | 3.01 (2.74 - 3.31)  (33.8) (443) | 2.70 (2.36 - 3.08)  (34.9) (221) |  |
|  |  |  |  |  |
| **Wrist** |  |  |  |  |
| <20.0 | 1.00 (0.87 - 1.15) ^d^  (20.4) (206) | 0.90 (0.74 - 1.09)  (20.7) (106) | 1.18 (0.99 - 1.40)  (20.0) (130) |  |
| 20.0-24.9 | 0.91 (0.87 - 0.95)  (24.5) (2024) | 0.87 (0.82 - 0.91)  (24.1) (1463) | 0.91 (0.86 - 0.97)  (24.1) (1205) | .42 ^c^ |
| 25.0-29.9 | 0.71 (0.68 - 0.75)  (28.7) (1627) | 0.73 (0.68 - 0.77)  (28.6) (1014) | 0.75 (0.69 - 0.81)  (28.6) (662) |  |
| *30+* | 0.59 (0.55 - 0.63)  (34.9) (797) | 0.58 (0.52 - 0.65)  (33.8) (327) | 0.55 (0.48- 0.64)  (34.9) (172) |  |
|  |  |  |  |  |
| **Hip** |  |  |  |  |
| <20.0 | 1.00 (0.89 - 1.12) ^d^  (20.4) (305) | 0.77 (0.64 - 0.93)  (20.7) (110) | 0.58 (0.47 - 0.72)  (20.0) (82) |  |
| 20.0-24.9 | 0.50 (0.47 - 0.52)  (24.5) (1444) | 0.38 (0.35 - 0.41)  (24.1) (700) | 0.32 (0.29 - 0.35)  (24.1) (475) | .77 ^c^ |
| 25.0-29.9 | 0.34 (0.32 - 0.36)  (28.7) (994) | 0.24 (0.21 - 0.26)  (28.6) (363) | 0.22 (0.19 - 0.25)  (28.6) (218) |  |
| *30+* | 0.23 (0.21 - 0.25)  (34.9) (406) | 0.19 (0.16 - 0.23)  (33.8) (119) | 0.14 (0.11- 0.19)  (34.9) (51) |  |

^a^ FCI = floating confidence interval

^b^ Number of women with a fracture in each category of BMI and strenuous activity

^c^ Likelihood ratio test for interaction between categories of BMI and activity

^d^ Referent group (RR = 1.0)

^e^ Adjusted for study region, age, socio-economic status, smoking, alcohol consumption, parity, use of hormone therapy, height, heart disease/thrombosis, diabetes mellitus, thyroid disease, and rheumatoid arthritis/osteoarthritis

| **eTable 4:** Sensitivity analyses for relative risk of ankle, wrist, and hip fractures in post-menopausal women according to BMI and physical activity | | | | | | | | | | | | |
| --- | --- | --- | --- | --- | --- | --- | --- | --- | --- | --- | --- | --- |
|  |  | **ANKLE FRACTURE** | | | **WRIST FRACTURE** | | | | **HIP FRACTURE** | | | |
|  |  | **Excluding the first three years of follow-up**  **RR (95% CI)** | **Excluding women with missing values for adjustment variables**  **RR (95% CI)** |  | | **Excluding the first three years of follow-up**  **RR (95% CI)** | **Excluding women with missing values for adjustment variables**  **RR (95% CI)** |  | | **Excluding the first three years of follow-up**  **RR (95% CI)** | **Excluding women with missing values for adjustment variables**  **RR (95% CI)** |  |
|  |  | n=5116 | n=6306 |  | | n=7646 | n=9060 |  | | n=4381 | n=4831 |  |
| **BMI (kg/m^2^)** |  |  |  |  | |  |  |  | |  |  |  |
| <20.0 (20.4) |  | 1.00 | 1.00 |  | | 1.00 | 1.00 |  | | 1.00 | 1.00 |  |
| 20.0-22.4 (22.6) |  | 1.43 (1.14 - 1.81) | 1.49 (1.20 - 1.84) |  | | 0.89 (0.80 - 1.00) | 0.94 (0.84 - 1.04) |  | | 0.62 (0.56 - 0.70) | 0.61 (0.55 - 0.68) |  |
| 22.5-24.9 (25.2) |  | 2.00 (1.60 - 2.50) | 2.08 (1.69 - 2.55) |  | | 0.81 (0.72 - 0.90) | 0.85 (0.76 - 0.94) |  | | 0.45 (0.40 - 0.50) | 0.44 (0.39 - 0.49) |  |
| 25.0-27.4 (27.7) |  | 2.48 (1.98 - 3.09) | 2.63 (2.14 - 3.23) |  | | 0.69 (0.62 - 0.78) | 0.74 (0.67 - 0.83) |  | | 0.37 (0.33 - 0.42) | 0.37 (0.33 - 0.41) |  |
| *27.5-29.9* (30.2) |  | 2.74 (2.19 - 3.43) | 2.96 (2.40 - 3.64) |  | | 0.64 (0.56 - 0.72) | 0.66 (0.59 - 0.74) |  | | 0.29 (0.25 - 0.33) | 0.28 (0.24 - 0.32) |  |
| *30+* (34.5) |  | 2.95 (2.36 - 3.69) | 3.25 (2.64 - 4.00) |  | | 0.54 (0.48 - 0.61) | 0.57 (0.51 - 0.64) |  | | 0.25 (0.21 - 0.28) | 0.22 (0.20 - 0.26) |  |
| *P-value (trend)* |  | < .001 | < .001 |  | | < .001 | < .001 |  | | < .001 | < .001 |  |
|  |  |  |  |  | |  |  |  | |  |  |  |
| **Strenuous Exercise** |  |  |  |  | |  |  |  | |  |  |  |
| Rarely/never (inactive) |  | 1.00 | 1.00 |  | | 1.00 | 1.00 |  | | 1.00 | 1.00 |  |
| At most once per week |  | 1.02 (0.96 - 1.09) | 1.03 (0.97 - 1.09) |  | | 0.96 (0.91 - 1.02) | 0.97 (0.92 - 1.02) |  | | 0.76 (0.71 - 0.82) | 0.74 (0.69 - 0.79) |  |
| More than once per week |  | 1.08 (1.00 - 1.16) | 1.06 (0.99 - 1.13) |  | | 1.01 (0.95 - 1.07) | 1.02 (0.96 - 1.06) |  | | 0.64 (0.58 - 0.69) | 0.62 (0.57 - 0.67) |  |
| *P-value (trend)* |  | .05 | .10 |  | | .96 | .76 |  | | < .001 | < .001 |  |
|  |  |  |  |  | |  |  |  | |  |  |  |
| **Any Exercise** |  | n=4547 | n=5638 |  | | n=6799 | n=8130 |  | | n=3805 | n=4232 |  |
| Rarely/never (inactive) |  | 1.00 | 1.00 |  | | 1.00 | 1.00 |  | | 1.00 | 1.00 |  |
| At most once per week |  | 1.05 (0.96 - 1.14) | 0.99 (0.92 - 1.08) |  | | 0.97 (0.90 - 1.05) | 0.94 (0.88 - 1.00) |  | | 0.80 (0.73 - 0.88) | 0.78 (0.72 - 0.86) |  |
| 2-3 times per week |  | 1.03 (0.94 - 1.13) | 0.99 (0.91 - 1.07) |  | | 0.98 (0.91 - 1.06) | 0.94 (0.88 - 1.00) |  | | 0.71 (0.64 - 0.78) | 0.67 (0.61 - 0.73) |  |
| More than 3 times per week |  | 1.13 (1.04 - 1.23) | 1.09 (1.01 - 1.18) |  | | 0.97 (0.91 - 1.04) | 0.93 (0.87 - 0.99) |  | | 0.77 (0.70 - 0.83) | 0.71 (0.66 - 0.77) |  |
| *P-value (trend)* |  | .005 | .01 |  | | .52 | .04 |  | | < .001 | < .001 |  |

^a^ Adjusted for study region, age, socio-economic status, smoking, alcohol consumption, parity, use of hormone therapy, height, heart disease/thrombosis, diabetes mellitus, thyroid disease, rheumatoid arthritis/osteoarthritis, and strenuous activity (for adjustment of BMI) or BMI (for adjustment of physical activity)

^b^ FCI = floating confidence interval

| **eTable 5:** Sensitivity analyses of hormone therapy for relative risk of ankle, wrist, and hip fractures in post-menopausal women according to BMI and physical activity | | | | | | | | | | | |
| --- | --- | --- | --- | --- | --- | --- | --- | --- | --- | --- | --- |
|  |  | **ANKLE FRACTURE** | | | **WRIST FRACTURE** | | | **HIP FRACTURE** | | | |
|  |  | **Restricted to current users of hormone therapy**  **RR (95% CI)** | **Restricted to women never using hormone therapy**  **RR (95% CI)** |  | **Restricted to current users of hormone therapy**  **RR (95% CI)** | **Restricted to women never using hormone therapy**  **RR (95% CI)** |  | | **Restricted to current users of hormone therapy**  **RR (95% CI)** | **Restricted to women never using hormone therapy**  **RR (95% CI)** |  |
|  |  | n=2066 | n=3334 |  | n=2149 | n=5639 |  | | n= 1213 | n=3152 |  |
| **BMI (kg/m^2^)** |  |  |  |  |  |  |  | |  |  |  |
| <20.0 (20.4) |  | 1.00 | 1.00 |  | 1.00 | 1.00 |  | | 1.00 | 1.00 |  |
| 20.0-22.4 (22.6) |  | 1.44 (1.01 - 2.06) | 1.37 (1.03 - 1.82) |  | 0.97 (0.78 - 1.21) | 0.97 (0.85 - 1.12) |  | | 0.62 (0.50 - 0.77) | 0.63 (0.55 - 0.73) |  |
| 22.5-24.9 (25.2) |  | 1.97 (1.40 - 2.78) | 2.04 (1.56 - 2.69) |  | 0.92 (0.74 - 1.14) | 0.85 (0.75 - 0.97) |  | | 0.44 (0.36 - 0.55) | 0.45 (0.39 - 0.51) |  |
| 25.0-27.4 (27.7) |  | 2.46 (1.75 - 3.48) | 2.52 (1.91 - 3.31) |  | 0.80 (0.64 - 1.00) | 0.74 (0.65 - 0.85) |  | | 0.39 (0.31 - 0.49) | 0.37 (0.32 - 0.42) |  |
| *27.5-29.9* (30.2) |  | 3.26 (2.30 - 4.62) | 2.71 (2.05 - 3.58) |  | 0.77 (0.61 - 0.97) | 0.69 (0.60 - 0.79) |  | | 0.30 (0.23 - 0.39) | 0.28 (0.24 - 0.33) |  |
| *30+* (34.5) |  | 3.62 (2.56 - 5.13) | 2.82 (2.14 - 3.72) |  | 0.71 (0.56 - 0.90) | 0.56 (0.49 - 0.65) |  | | 0.29 (0.22 - 0.38) | 0.22 (0.19 - 0.25) |  |
| *P-value (trend)* |  | <.001 | < .001 |  | < .001 | < .001 |  | | < .001 | < .001 |  |
|  |  |  |  |  |  |  |  | |  |  |  |
| **Strenuous Exercise** |  |  |  |  |  |  |  | |  |  |  |
| Rarely/never (inactive) |  | 1.00 | 1.00 |  | 1.00 | 1.00 |  | | 1.00 | 1.00 |  |
| At most once per week |  | 1.01 (0.92 - 1.12) | 1.04 (0.96 - 1.12) |  | 1.00 (0.90 - 1.11) | 0.96 (0.90 - 1.02) |  | | 0.83 (0.72 - 0.94) | 0.71 (0.65 - 0.77) |  |
| More than once per week |  | 1.06 (0.95 - 1.19) | 1.07 (0.98 - 1.17) |  | 1.05 (0.94 - 1.18) | 1.00 (0.93 - 1.07) |  | | 0.63 (0.53 - 0.74) | 0.61 (0.55 - 0.67) |  |
| *P-value (trend)* |  | .35 | .14 |  | .39 | .71 |  | | < .001 | < .001 |  |
|  |  |  |  |  |  |  |  | |  |  |  |
| **Any Exercise** |  | n=1834 | n=2991 |  | n=1939 | n=5040 |  | | n=1063 | n=2746 |  |
| Rarely/never (inactive) |  | 1.00 | 1.00 |  | 1.00 | 1.00 |  | | 1.00 | 1.00 |  |
| At most once per week |  | 1.05 (0.92 - 1.21) | 0.93 (0.84 - 1.04) |  | 0.85 (0.74 - 0.98) | 0.92 (0.84 - 1.00) |  | | 0.78 (0.64 - 0.93) | 0.80 (0.71 - 0.89) |  |
| 2-3 times per week |  | 1.03 (0.89 - 1.19) | 0.96 (0.86 - 1.08) |  | 0.84 (0.73 - 0.96) | 0.93 (0.86 - 1.02) |  | | 0.78 (0.65 - 0.94) | 0.63 (0.56 - 0.70) |  |
| More than 3 times per week |  | 1.17 (1.02 - 1.33) | 1.04 (0.94 - 1.15) |  | 0.92 (0.81 - 1.04) | 0.92 (0.85 - 1.00) |  | | 0.86 (0.73 - 1.01) | 0.67 (0.60 - 0.74) |  |
| *P-value (trend)* |  | .03 | .22 |  | .38 | .10 |  | | .19 | < .001 |  |

^a^ Adjusted for study region, age, socio-economic status, smoking, alcohol consumption, parity, height, heart disease/thrombosis, diabetes mellitus, thyroid disease, rheumatoid arthritis/osteoarthritis, and strenuous activity (for adjustment of BMI) or BMI (for adjustment of physical activity)
